# Knowledge, attitude, and practice toward photoaging in the Chinese population: a cross-sectional study

**DOI:** 10.1038/s41598-024-55691-5

**Published:** 2024-03-02

**Authors:** Yaoying Li, Tianxing Hu, Xiaoqin Xia, Lan Ge

**Affiliations:** Department of Dermatology, Southwest Hospital, Army Military Medical University, Chongqing, 400038 China

**Keywords:** Photoaging, Knowledge, Attitude, Practice, Questionnaire, Cross-sectional study, Health care, Medical research

## Abstract

To investigate the knowledge, attitude, and practice (KAP) of photoaging in the Chinese population. This web-based cross-sectional study was conducted between January 2023 and March 2023 among the Chinese population aged 18–80 years old. Participants’ knowledge, attitude, and practice toward photoaging were collected through a self-administered questionnaire. A total of 830 questionnaires were collected, with 826 valid questionnaires and an efficiency rate of 99.52%. There were 274 (33.17%) males and 532 (64.41%) aged 31–51 years old. The average knowledge, attitude, and practice scores were 7 (4, 9) (possible range 0–12), 31.5 (28, 34) (possible range 8–40), and 33 (24, 42) (possible range 11–55), respectively, indicating poor knowledge, good attitude, and moderate practice. Spearman correlation analysis showed that knowledge was negatively correlated with attitude (r = − 0.111, P < 0.05) and practice (r = − 0.113, P < 0.05), and attitude was positively correlated with practice (r = 0.992, P < 0.05). The multivariable linear regression model showed that for each point increase in attitude score, the practice score increased by 2.96 points (β = 2.96, 95% CI 2.91–3.01, P < 0.001). The Chinese population has poor knowledge, good attitude, and moderate practice toward photoaging. A good attitude toward photoaging would lead to good practice, and more outreach and education for the Chinese population might be needed.

## Introduction

The skin is responsible for several physiologic functions, including protection from physical, chemical, and microbial elements, prevention of evaporative water loss, thermoregulation through sweating and vascular dilation or constriction, mediating the sense of touch, and serving as a sentry for immune surveillance^1^. Skin aging can be divided into unavoidable intrinsic aging and avoidable extrinsic aging^2^. Intrinsic (chronologic) aging is dominated by fine lines and increased skin laxity (due to fat atrophy), gravity-induced soft tissue redistribution, and reduction of facial skeletal support related to bone resorption^1,3^. The clinical signs of chronological aging also include benign vascular formations such as cherry angiomas and benign overgrowths such as seborrheic keratoses^1,3^. The extrinsic factors in skin aging include ultraviolet light and environmental damage (e.g., pollution, cigarette smoke, and various toxins)^4^.

Photoaging accounts for more than 80% of facial aging^5^. Changes from exposure to solar ultraviolet rays are superimposed on intrinsic processes of aging, with structural changes in the skin, including decreased cellular DNA quality and altered collagen structure^3^. Hence, long-term exposure to UV radiation causes skin tissue changes, resulting in roughness, thickening, and wrinkles, as well as discoloration and acne, which are the main causes of accelerated photoaging. UVA radiation is the main culprit of photoaging and skin cancer^6^. Severe photoaging can lead to benign and malignant skin tumors such as solar keratosis and melanoma^7^. The most important cause of photoaging is UV radiation, which is time-dependent and dose-dependent. Therefore, the simplest and most effective way to combat photoaging is to reduce skin exposure to UV radiation by using long clothes, shades, and sunscreen products^8^.

Due to cultural differences between China and the West, Chinese women prefer lighter skin tones, and tanning behaviors are less common^9,10^, while more and more Chinese people are becoming concerned about skin aging as the economy grows. There are few relevant studies on sun exposure and protection behaviors in China. Previous findings suggest a deficit in sun protection knowledge, attitude, and practice in China^11^, but no studies related to photoaging could be found.

A knowledge, attitude, and practice (KAP) survey is a quantitative method that provides quantitative and qualitative data about the misconceptions and deficiencies of a specific population toward a specific subject^12,13^. Understanding the Chinese population’s KAP toward photoaging may be the cornerstone of designing successful interventions to identify knowledge deficits and promote anti-photoaging behaviors in the Chinese population. Therefore, this study aimed to investigate the KAP of photoaging in the Chinese population.

## Results

### Demographic characteristics

**Figure 1:**
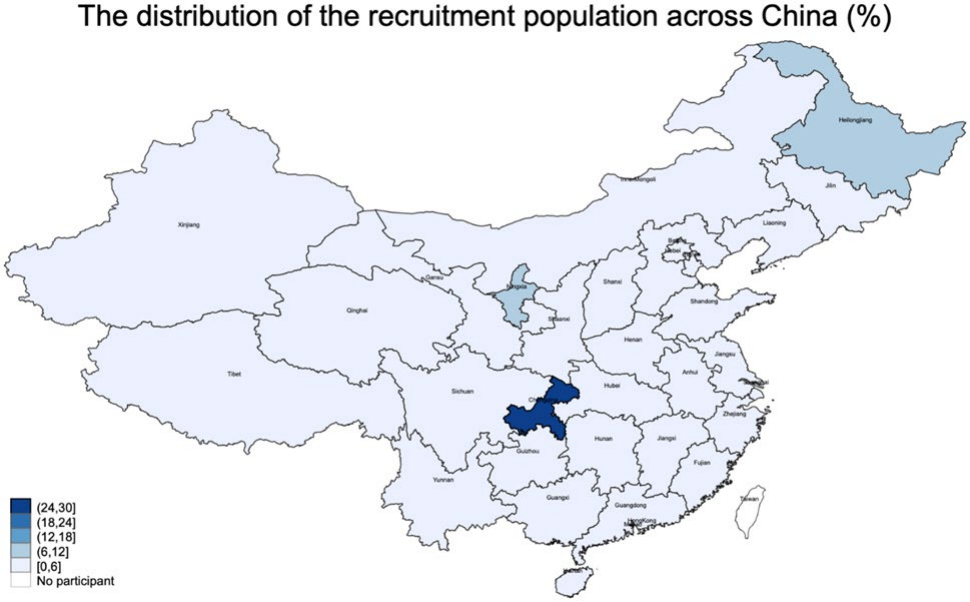
The distribution of the recruitment population in each region. *This map does not contain 1 foreign participant, accounting for 0.12%; the islands around China are not shown in this map.

A total of 830 questionnaires were collected, with 826 valid questionnaires and an efficiency rate of 99.52%. Of the participants, 274 (33.17%) were male, 532 (64.41%) were 31–51 years old, 705 (85.35%) lived in urban areas, 120 (14.53%) had an education level of high school or below, 175 (21.19%) were general staff, 281 (34.02%) had a household monthly per capita income of > 10,000 RMB, and 190 (23.00%) were unmarried. The average knowledge, attitude, and practice scores were 7 (4, 9) (possible range 0–12), 31.5 (28, 34) (possible range 8–40), and 33 (24, 42) (possible range 11–55), respectively, indicating poor knowledge, good attitude, and moderate practice. There were significant differences in knowledge among participants by gender (P < 0.001), occupation (P = 0.005); in attitude by gender (P < 0.001), age (P < 0.001), residence (P = 0.001), household monthly per capita income level (P < 0.001), and marriage status (P < 0.001); and in practice scores by gender (P < 0.001), age (P < 0.001), residence (P = 0.001), household monthly per capita income level (P < 0.001), and marriage status (P < 0.001) (Table 1). The participants were located almost throughout China (Fig. 1).

**Table 1:** Baseline characteristics and KAP scores.

Variable	N = 826	Knowledge median (IQR)	P	Attitude median (IQR)	P	Practice median (IQR)	P
Total		7 (4, 9)		31.5 (28, 34)		33 (24, 42)	
Gender			< 0.001		< 0.001		< 0.001
Male	274 (33.17)	6 (3, 8)		32 (30, 35)		35 (30, 44)	
Female	552 (66.83)	7 (5, 9)		30 (28, 34)		31 (23, 42)	
Age, year			0.086		< 0.001		< 0.001
≤ 30	227 (27.48)	7 (4, 9)		33 (28, 36)		37 (27, 49)	
31–50	532 (64.41)	7 (5, 9)		31 (28, 34)		32 (23, 40.5)	
≥ 51	67 (8.11)	6 (4, 8)		32 (28, 34)		33 (27, 42)	
Residence			0.319		0.001		0.001
Urban areas	705 (85.35)	7 (4, 9)		31 (28, 34)		32 (23, 42)	
Non-urban areas	121 (14.65)	7 (4, 9)		33 (30, 35)		37 (30, 46)	
Education			0.125		0.498		0.430
High school and below	120 (14.53)	6 (4, 9)		32 (28, 34)		33 (25, 42)	
College and Undergraduate	541 (65.50)	7 (5, 9)		32 (28, 34)		33 (26, 43)	
Master’s degree and above	165 (19.98)	7 (4, 9)		31 (28, 34)		33 (21, 42)	
Occupation			0.005		0.619		0.570
Professional and technical staff	271 (32.81)	8 (5, 9)		32 (28, 35)		33 (22, 44)	
General staff	175 (21.19)	7 (4, 9)		31 (28, 34)		31 (26, 42)	
Other	380 (46.00)	7 (4, 9)		32 (28, 34)		33 (25, 42)	
Household monthly per capita income, RMB			0.504		< 0.001		< 0.001
≤ 5000	239 (28.93)	6 (4, 9)		33 (29, 35)		36 (29, 46)	
5001–10,000	306 (37.05)	7 (4, 9)		31 (28, 34)		32 (24, 41)	
≥ 10,001	281 (34.02)	7 (5, 9)		31 (28, 34)		32 (22, 39)	
Marriage status			0.061		< 0.001		< 0.001
Unmarried	190 (23.00)	7 (4, 9)		33 (28, 36)		37 (27, 52)	
Married	636 (77.00)	7 (5, 9)		31 (28, 34)		32 (23, 41)	

The P-values refer to the comparison of the values of the difference variables (e.g., male vs. female) for each KAP dimension.

### Knowledge, attitude, and practice dimensions

**Figure 2:**
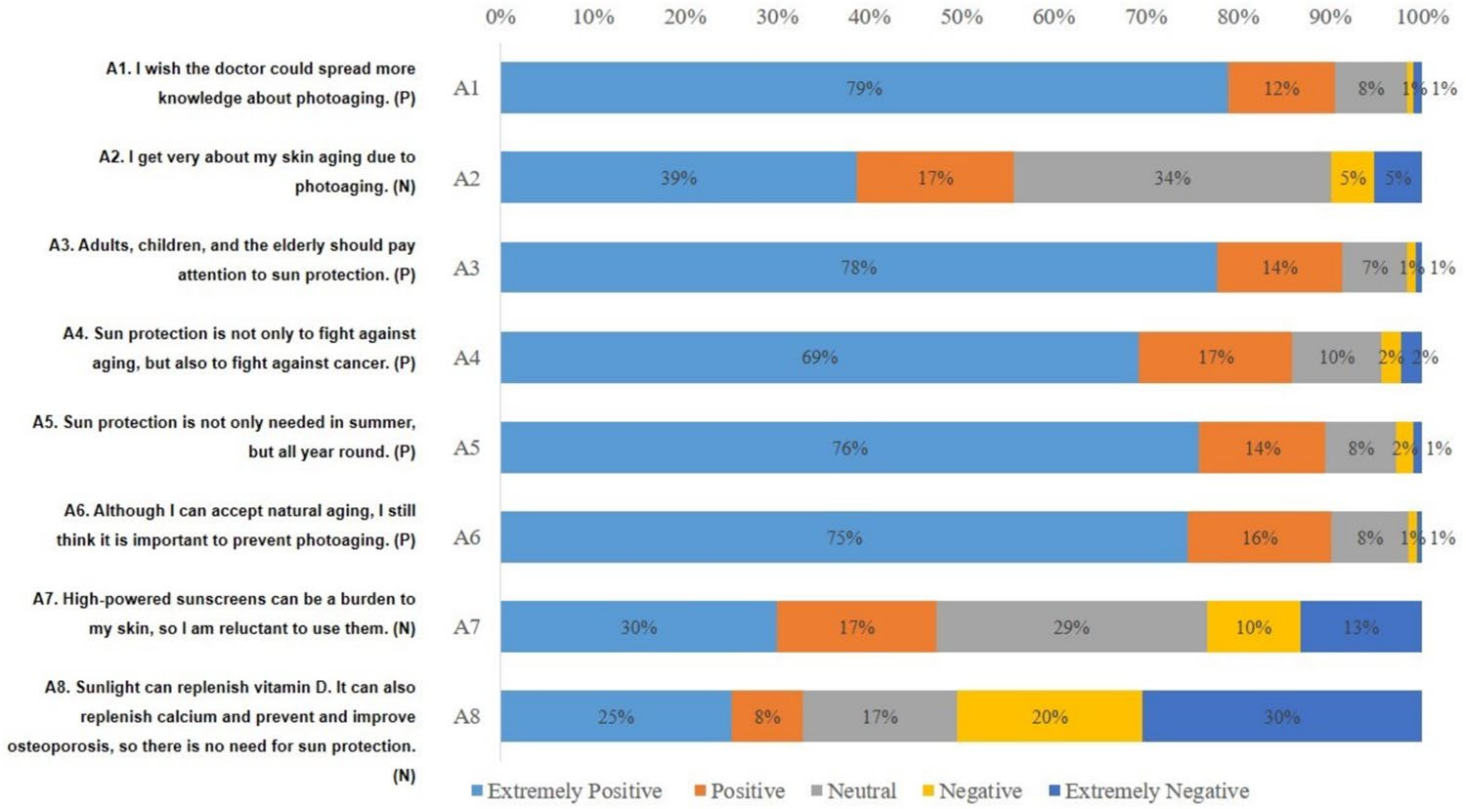
The score distribution of the “attitude” dimension. *P refers to a positive formulation of the topic, N refers to a negative formulation of the topic, and what is shown in this figure is already the result adjusted for the assignment.

**Figure 3:**
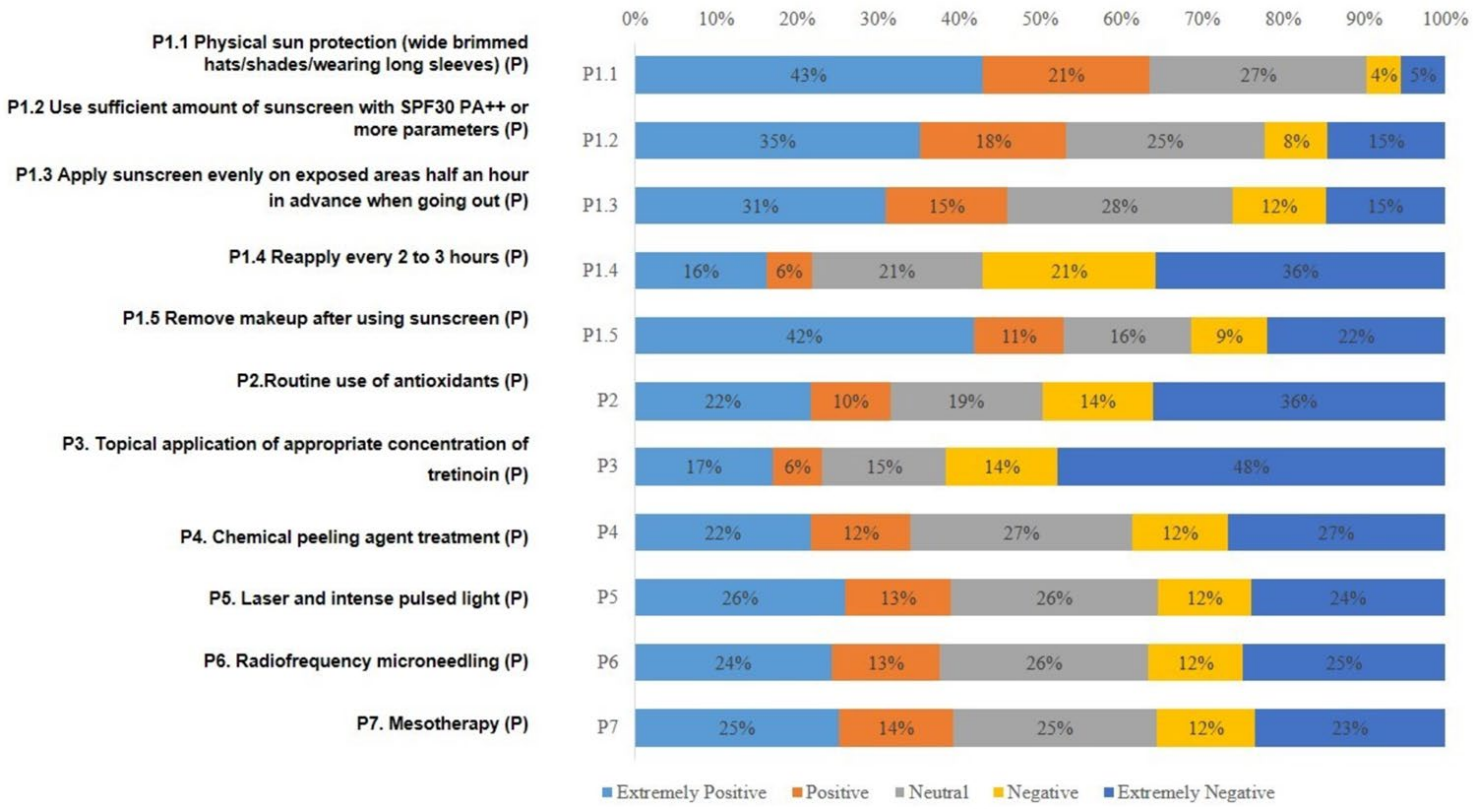
The score distribution of the “practice” dimension. *P refers to a positive formulation of the topic, N refers to a negative formulation of the topic, and what is shown in this figure is already the result adjusted for the assignment.

In knowledge dimension, the highest scoring question was K1 (Skin aging encompasses both intrinsic and extrinsic factors. Intrinsic aging refers to the natural aging of the skin due to the decline in cellular metabolism associated with the aging process, resulting in the skin aging naturally. Extrinsic aging, on the other hand, is attributed to environmental factors from the external surroundings that contribute to skin aging.), with a correct rate of 86.80%. The lowest scoring question was K6 (The SPF indicated on sunscreen reflects its protective ability against UVB. Generally, a higher SPF number suggests an extended duration of protection against sunburn. [False]), with a correct rate of 8.11% (Table 2). In attitude dimension, A8 (Sunlight can replenish vitamin D. It can also replenish calcium, prevent and improve osteoporosis, so there is no need for sun protection. [Negative]) had the most people choosing very negative, followed by A7 (High-powered sunscreens can be a burden to my skin, so I am reluctant to use them. [Negative]) and A2 (I get very anxious when I think of my skin aging due to photoaging. [Negative]) (Fig. 2). In practice dimension, P3 (Topical application of appropriate concentration of tretinoin [Positive]) had the most people choosing very negative, followed by P2 (Routine use of antioxidants [Positive]) and P1.4 (Reapply every 2 to 3 h [Positive]) (Fig. 3).

**Table 2:** Distribution of knowledge dimension.

Knowledge	N (%)	
	Correct answer	Wrong answer/unclear
K1: Skin aging encompasses both intrinsic and extrinsic factors. Intrinsic aging refers to the natural aging of the skin due to the decline in cellular metabolism associated with the aging process, resulting in the skin aging naturally. Extrinsic aging, on the other hand, is attributed to environmental factors from the external surroundings that contribute to skin aging. (True)	717 (86.80)	109 (13.20)
K2: The most common cause of skin aging is photoaging, with the primary factor contributing to photoaging being exposure to ultraviolet radiation. (True)	611 (73.97)	215 (26.03)
K3: Photoaging can result in skin roughness, thickening, dilated blood vessels, wrinkles, pigmentation, and even the development of cancer in exposed areas such as the face and hands. (True)	594 (71.91)	232 (28.09)
K4: The UVA and UVB components in ultraviolet radiation both pose harm to the skin, causing discomfort especially in summer. (False) ^a^	79 (9.56)	747 (90.44)
K5: UVB is more likely to induce non-melanoma skin cancer and actinic keratosis than UVA. (True)	449 (54.36)	377 (45.64)
K6: The SPF indicated on sunscreen reflects its protective ability against UVB. Generally, a higher SPF number suggests an extended duration of protection against sunburn. (False)^b^	67 (8.11)	759 (91.89)
K7: The PA value of sunscreen reflects the protection ability against UVA; the more “ + ” after PA, the stronger the protection against UVA. (True)	572 (69.25)	254 (30.75)
K8: To ensure effective protection, it is essential to apply an adequate amount of sunscreen, approximately 2 mg per square centimeter. (True)	452 (54.72)	374 (45.28)
K9: Adopting a scientifically balanced diet can also contribute to sun protection. Certain foods are photosensitive, and the consumption of "photosensitive foods" may lead to sun-related dermatitis. (True)	581 (70.34)	245 (29.66)
K10: Darker, thicker clothing is more effective in protecting against the sun. (True)	420 (50.85)	406 (49.15)
K11: Topical application of retinoic acid can alleviate fine lines and uneven pigmentation associated with photoaging, regardless of the concentration used. (False)	229 (27.72)	597 (72.28)
K12: Chemical peels, dermabrasion, photodynamic therapy, laser treatments, intense pulsed light, and topical, injectable fillers can be used to improve photoaging. (True)	436 (52.78)	390 (47.22)

^a^False refers to the statement that the question stem is wrong.

^b^According to Chinese regulations, SPF labeling shall not be higher than 50, while some countries allow SPF values higher than 50.

The specific levels of KAP differed between participants of different genders (P < 0.05) except for A2 (Anxiety due to skin aging caused by photoaging) and A8 (Sunlight can replenish vitamin D. It can also replenish calcium and prevent and improve osteoporosis, so sun protection is not necessary); by household monthly per capita income level at K1-K3 (the basics of photoaging) (P = 0.022), A4 (Sun protection is not only to be anti-aging but also to fight cancer) (P = 0.026), A5 (Sun protection is needed not only in summer, but all year round) (P < 0.001), A6 (Although I can accept natural aging, I still think it is important to prevent photoaging) (P < 0.001), and P1.1–P1.5 (Reduce skin exposure and use sunscreen products) (P < 0.001), P2 (Routine use of antioxidants) (P = 0.011) and P4 (Chemical peel agent treatment) (P < 0.001), P5 (Laser and intense pulsed light) (P < 0.001), P6 (Radiofrequency microneedling) (P < 0.001) and P7 (Mesotherapy) (P < 0.001); by education levels at A6 (Although I can accept natural aging, I still think it is important to prevent photoaging) (P = 0.030); by residence type at A8 “Sunlight can replenish vitamin D. It can also replenish calcium and prevent and improve osteoporosis, so sun protection is not necessary) (P = 0.002), P1.1–P1.5 (Reduce skin exposure and use sunscreen products) (P = 0.004), P2 (Routine use of antioxidants) (P = 0.008), P4 (Chemical peel agent treatment) (P = 0.010), P5 (Laser and intense pulsed light) (P < 0.001), P6 (Radiofrequency microneedling) (P = 0.002) and P7 (Mesotherapy) (P = 0.022), and there were significant differences at age except for K4-K10 (UV and Sun Protection) (Supplementary Table S1).

### Spearman correlation analysis

Spearman correlation analysis showed that knowledge was negatively correlated with attitude (r = − 0.111, P < 0.05) and practice (r = − 0.113, P < 0.05), and attitude was positively correlated with practice (r = 0.992, P < 0.05) (Table 3).

**Table 3:** Spearman correlation analysis.

	Knowledge	Attitude	Practice
Knowledge	1		
Attitude	− 0.111*	1	
Practice	− 0.113*	0.992*	1

*P < 0.05.

### The univariate and multivariate linear regression analysis

The univariate and multivariate linear regression analysis showed that for each point increase in attitude score, the practice score increased by 2.96 points (β = 2.96, 95% CI 2.91–3.01, P < 0.001) (Table 4).

**Table 4:** Multivariable linear regression analysis of the factors influencing practice.

Variable	Univariate linear regression		Multivariate linear regression	
	β (95% CI)	P	β (95% CI)	P
Knowledge	− 0.54 (− 0.84, − 0.24)	< 0.001	− 0.03 (− 0.11, 0.03)	0.314
Attitude	2.96 (2.91, 3.01)	< 0.001	2.96 (2.91, 3.01)	< 0.001
Gender				
Male	Ref.		Ref.	
Female	− 4.37 (− 6.13, − 2.60)	< 0.001	0.15 (− 0.20, 0.60)	0.486
Age, years				
≤ 30	Ref.		Ref.	
31–50	− 4.94 (− 6.83, − 3.05)	< 0.001	0.01 (− 0.58, 0.61)	0.966
≥ 51	− 2.93 (− 6.25, 0.38)	0.083	− 0.54 (− 1.44, 0.36)	0.239
Residence type				
Urban areas	Ref.		Ref.	
Non-urban areas	3.94 (1.57, 6.30)	0.001	0.26 (− 0.32, 0.85)	0.375
Education level				
High school and below	Ref.			
College and Undergraduate	− 0.97 (− 3.42, 1.46)	0.432		
Master’s degree and above	− 1.70 (− 4.60, 1.19)	0.25		
Occupation type				
Professional and technical staff	Ref.			
General staff	− 0.26 (− 2.61, 2.08)	0.825		
Other	0.64 (− 1.28, 2.56)	0.512		
Household monthly per capita income, RMB				
≤ 5000	Ref.		Ref.	
5001–10,000	− 4.50 (− 6.56, − 2.44)	< 0.001	0.12 (− 0.38, 0.63)	0.627
≥ 10,001	− 4.98 (− 7.08, − 2.88)	< 0.001	0.27 (− 0.24, 0.80)	0.3
Marriage status				
Unmarried	Ref.		Ref.	
Married	− 5.64 (− 7.60, − 3.67)	< 0.001	− 0.30 (− 0.92, 0.31)	0.33

## Discussion

This study found that public in China had poor knowledge, good attitude, and moderate practice toward photoaging, and good attitude leads to good practice, proving the need for more popularization of photoaging knowledge among the public in China, which may provide a reference for future targeted educational efforts.

The results are consistent with other studies in which knowledge and awareness of the harmful effects of sun exposure are widespread in China, and increased awareness of the risks does not necessarily lead to good behavior^11,14^. According to the announcement of the State Food and Drug Administration (SFDA)^15^, the Sun Protection Factor (SPF) value should not be higher than 50. It may not be known by many people and therefore got a very low correct rate in K6 (8.11%), which may make consumers think that foreign products have better sun protection when choosing product. The K4 also got a low correct rate (9.56%). According to the different wavelengths, the ultraviolet rays in sunlight are mainly divided into UVA, UVB, and UVC; UVB will increase a lot in summer, and although the wavelength is shorter than UVA and will not invade the dermis or even the subcutaneous tissue area, it is highly mutagenic and can cause skin redness, molting, itching, dryness, and keratin damage^16–18^. These information should be disseminated among the general public.

One study suggested that there is little consensus about sun exposure among physicians^19^, which may lead to confusion about the relationship between sun exposure behaviors, sun protection behaviors, and vitamin D, as reflected consistently in A8. Daily and/or recreational use of daily broad-spectrum sunscreens with high UVA protection does not impair vitamin D status in healthy individuals^20^, which requires more education.

Tanning is only one manifestation of UV damage to the skin, but photoaging is actually much more than what is visible to the naked eye; it is more frightening than natural aging^21^. Retinoic acid promotes collagen synthesis in photoaging skin and has been widely used in cosmetics and dermatological treatments^22,23^. UV radiation causes severe oxidative stress that stimulates skin aging, leading to melanogenesis and wrinkle formation, and antioxidants are considered an important strategy for treating skin photoaging^24–26^. In this study, however, the application of retinoids and antioxidants in the Chinese population was not positive, which may need to be improved.

The results of this study was somewhat different from other studies^27,28^, in which men, those in non-urban areas, and those with household monthly per capita income ≤ 5000 had higher attitude and practice scores. With the rapid development of economic level, there is a growing awareness of the harmful effects of sunlight on the skin and the importance of sun protection, which may be facilitated by the media and cosmetic companies’ publicity and advertising.

A dissociation was observed in the present study regarding the correlations between knowledge, attitude and practice. Indeed, it is generally acknowledged that better knowledge will translate into better attitude and practice^29,30^. Still, such a dissociation has been reported in medical students toward geriatric education, in whom improved knowledge did not translate into a better attitude^31^. Still, in the present study, this population had poor knowledge, but good attitude and moderate practice. It could be due to the television, radio, and internet advertisements promoting skin protection and influencing the attitude and practice of the population toward photoaging and photoprotection, but lack of professional and accurate information, or insufficient public attention to relevant knowledge. Moreover, some advertisements are too short to allow any knowledge transmission and only insist on a specific behavior. Another explanation might be that a lower level of knowledge led to a lower acknowledgment of the possible barriers to photoaging protection^32^. People with higher knowledge about a specific condition can be more fatalistic regarding that condition^33^. Still, that specific relationship will have to be investigated in more detail.

There are several limitations to this study. Although the researchers were spread across China, there may still be selection bias, and the need for cell phone use for online questionnaire respondents might exclude many older people. In addition, for questionnaire-type studies, there may be social desirability bias^34^ in which although we designed incorrect question stems in the knowledge dimension, participants may show a learned characteristic by answering affirmatively to a question that is unclear in itself, thus making the knowledge score high.

In conclusion, this study suggest that the Chinese public’s KAP toward photoaging needs to be improved, especially in terms of knowledge. A good attitude might lead to good practice. In the future, the Chinese population will be educated, and by promoting their good attitude, good practice will be obtained.

## Methods

### Study design and participants

This web-based cross-sectional study was conducted between January 2023 and March 2023. The participants were required to be ≥ 18 years of age, excluding questionnaires with incomplete responses. The study was approved by the ethics committee of the Southwest Hospital of Army Medical University approved this study (NO.KY2023010). Informed consent was obtained from all study participants.

### Procedures

A self-administered questionnaire was designed for the study based on the WHO KAP development guideline^35^, Treatment of Photoaging^36^, and Skin cancer and photoprotection in people of color: A review and recommendations for physicians and the public^37^. This questionnaire contained four parts, including (1) participant demographic characteristics, including gender, age, residence type, education level, occupational type, household monthly per capita income, and marital status, (2) the knowledge dimension, which consisted of 12 questions about photoaging, with a score of 1 for correct answers and 0 for incorrect or unclear answers, ranging from 0 to 12, (3) the attitude dimension, which consisted of eight questions, all using a five-point Likert scale, ranging from “extremely positive” (5 points) to “extremely negative” (1 point), with a score of 8–40 points, and 4) the practice dimension, which consisted of 11 questions, also using a five-point Likert scale, ranging from “always” (5 points) to “never” (1 point), with a score of 11–55 points. Higher scores indicated more favorable KAP. A score greater than 75% was considered good, in the range of 60–75% was considered moderate, and less than 60% was considered poor.

After the questionnaire was designed and modified with reference to comments made by three experts (two dermatologists and one epidemiologist), a small pretest (134 copies) was conducted before the final questionnaire was formally released; the Cronbach’s α was 0.913, and the KMO was 0.852, suggesting a high degree of consistency within the questionnaire. To ensure nationwide participation, we enlisted local volunteers from the seven sub-regions of China, namely Northeast, East, North, Central, South, Southwest, and Northwest. These volunteers were trained, briefed on the study’s purpose and the necessary precautions, and deployed using the convenience sampling method for placement. The Questionnaire Star (Changsha Ranxing Information Technology Co., Ltd.) was used to construct the online questionnaire and generate a Quick Response (QR) code. The participants scanned the sent QR code through WeChat to log in and fill out the questionnaire. To ensure the quality and integrity of the questionnaire results, the participants had to authenticate when answering, each IP address could only be submitted once, all items were mandatory, and a verification code had to be entered manually when submitting. Members of the researchers’ team checked all questionnaires for completeness, internal coherence, and reasonableness.

### Sample size

The sample size was calculated using the formula for cross-sectional studies: α = 0.05, ${\text{n}}={\left(\frac{{Z}_{1-\alpha /2}}{\delta }\right)}^{2}\times p\times \left(1-p\right)$ where ${Z}_{1-\alpha /2}$ = 1.96 when α = 0.05, the assumed degree of variability of $p$ = 0.5 maximizes the required sample size, and δ is an admissible error (which was 5% here). The theoretical sample size was 480, which includes an extra 20% to allow for subjects lost during the study.

### Statistical analysis

Stata 17.0 (Stata Corp-College Station-TX-USA) was used to perform the statistical and descriptive analyses of the respondents’ demographic characteristics and KAP scores. Skewness and kurtosis tests were used to assess the normality. Continuous data with a normal distribution were described as means ± standard deviation (SD) and analyzed using Student’s t-test; otherwise, they were presented as medians (interquartile range, IQR) and analyzed using the Wilcoxon rank-sum test. Spearman correlation analysis was used to evaluate the correlation between the three dimensions. Univariate and multivariate linear regression analysis was used to identify risk factors on practice. Variance inflation factor (VIF) was used to test for the co-linearity of the variables. All variables included in the final model had a VIF < 3^38^. Two-sided P < 0.05 was considered statistically significant.

### Ethics approval and consent to participate

The study was carried out after the protocol was approved by the ethics committee of the Southwest Hospital of Army Medical University. I confirm that all methods were performed in accordance with the relevant guidelines. All procedures were performed in accordance with the ethical standards laid down in the 1964 Declaration of Helsinki and its later amendments. Informed consent was obtained from all study participants.

### Supplementary Information

Supplementary Table S1.

## Data Availability

All data generated or analyzed during this study are included in this published article.
