# Supplementary material for: Knowledge, attitude, and practice toward photoaging in the Chinese population: a cross-sectional study
Source: Sci Rep. 2024 Mar 2;14:5196. doi: 10.1038/s41598-024-55691-5 (PMC10908786; doi:10.1038/s41598-024-55691-5)
Supplement: Supplementary file 1 — Supplementary Table S1. [file 41598_2024_55691_MOESM1_ESM.docx]

**Supplementary Table S1.** Specific differences in KAP scores for participants with different demographic characteristics

| **Factor or Statement** | Gender | | | Household monthly per capita income level | | | | Education level | | | |
| --- | --- | --- | --- | --- | --- | --- | --- | --- | --- | --- | --- |
|  | Male | Female | P | ≤ 5000 | 5001-10000 | ≥ 10001 | P | High school and below | College & Undergraduate | Master's degree and above | P |
| **Knowledge** |  |  |  |  |  |  |  |  |  |  |  |
| The basics of photoaging (K1-K3) | 2.07 ± 1.10 | 2.45 ± 0.88 | <0.001 | 2.16 ± 1.06 | 2.39 ± 0.91 | 2.40 ± 0.95 | 0.022 | 2.18 ± 0.94 | 2.35 ± 0.98 | 2.36 ± 0.98 | 0.218 |
| UV and Sun Protection (K4-K10) | 2.59 ± 1.89 | 3.46 ± 1.54 | <0.001 | 3.00 ± 1.76 | 3.16 ± 1.73 | 3.33 ± 1.64 | 0.253 | 2.98 ± 1.79 | 3.24 ± 1.71 | 3.10 ± 1.64 | 0.269 |
| Methods to improve photoaging (K11-K12) | 0.69 ± 0.67 | 0.86 ± 0.67 | <0.001 | 0.76 ± 0.67 | 0.79 ± 0.68 | 0.85 ± 0.67 | 0.423 | 0.73 ± 0.72 | 0.83 ± 0.66 | 0.76 ± 0.68 | 0.229 |
| **Attitude** |  |  |  |  |  |  |  |  |  |  |  |
| Anxiety due to skin aging caused by photoaging (A2) | 2.20 ± 1.17 | 2.21 ± 1.16 | 0.846 | 2.26 ± 1.15 | 2.21 ± 1.13 | 2.16 ± 1.20 | 0.925 | 2.13 ± 1.09 | 2.25 ± 1.17 | 2.12 ± 1.18 | 0.308 |
| Sun protection is not only to be anti-aging but also to fight cancer (A4) | 4.64 ± 0.79 | 4.41 ± 0.97 | <0.001 | 4.63 ± 0.74 | 4.43 ± 0.94 | 4.43 ± 1.02 | 0.026 | 4.60 ± 0.82 | 4.46 ± 0.94 | 4.48 ± 0.91 | 0.332 |
| Sun protection is needed not only in summer but all year round (A5) | 4.81 ± 0.51 | 4.52 ± 0.87 | <0.001 | 4.78 ± 0.61 | 4.57 ± 0.73 | 4.53± 0.94 | <0.001 | 4.73 ± 0.59 | 4.62 ± 0.78 | 4.52 ± 0.91 | 0.093 |
| Although I can accept natural aging, I still think it is important to prevent photoaging (A6) | 4.74 ± 0.56 | 4.57 ± 0.79 | <0.001 | 4.79 ± 0.57 | 4.57 ± 0.72 | 4.56 ± 0.83 | <0.001 | 4.76 ± 0.50 | 4.63 ± 0.76 | 4.53 ± 0.75 | 0.030 |
| Sunlight can replenish vitamin D. It can also replenish calcium and prevent and improve osteoporosis, so sun protection is not necessary (A8) | 3.39± 1.59 | 3.16 ± 1.55 | 0.051 | 3.45 ± 1.57 | 3.20 ± 1.52 | 3.09 ± 1.59 | 0.107 | 3.18 ± 1.66 | 3.22 ± 1.54 | 3.32 ± 1.57 | 0.744 |
| **Practice** |  |  |  |  |  |  |  |  |  |  |  |
| Reduce skin exposure and use sunscreen products (P1.1-P1.5) | 17.85 ± 5.53 | 16.09 ± 5.82 | <0.001 | 18.14 ± 5.35 | 16.16 ± 5.74 | 15.99 ± 5.98 | <0.001 | 17.33 ± 5.07 | 16.68 ± 5.68 | 16.19 ± 6.55 | 0.265 |
| Routine use of antioxidants (P2) | 2.89 ± 1.56 | 2.57 ± 1.56 | 0.005 | 2.98 ± 1.56 | 2.55 ± 1.55 | 2.56 ± 1.55 | 0.011 | 2.73 ± 1.45 | 2.67 ± 1.57 | 2.67 ± 1.62 | 0.938 |
| Topical application of appropriate concentrations of retinoic acid (P3) | 2.53 ± 1.54 | 2.20 ± 1.51 | 0.003 | 2.54 ± 1.57 | 2.17 ± 1.46 | 2.26 ± 1.53 | 0.067 | 2.19 ± 1.46 | 2.30 ± 1.50 | 2.44 ± 1.65 | 0.386 |
| Chemical peel agent treatment (P4) | 3.23 ± 1.41 | 2.74 ± 1.48 | <0.001 | 3.26 ± 1.41 | 2.77 ± 1.42 | 2.74 ± 1.54 | <0.001 | 3.05 ± 1.38 | 2.89 ± 1.48 | 2.84 ± 1.55 | 0.470 |
| Laser and intense pulsed light (P5) | 3.39 ± 1.41 | 2.89 ± 1.52 | <0.001 | 3.40 ± 1.43 | 2.94 ± 1.47 | 2.90 ± 1.55 | <0.001 | 3.11 ± 1.45 | 3.07 ± 1.50 | 2.99 ± 1.55 | 0.767 |
| Radiofrequency microneedling (P6) | 3.35 ± 1.38 | 2.84 ± 1.52 | <0.001 | 3.34 ± 1.44 | 2.94 ± 1.47 | 2.80 ± 1.52 | <0.001 | 3.09 ± 1.46 | 3.02 ± 1.49 | 2.93 ± 1.52 | 0.646 |
| Mesotherapy (P7) | 3.36 ± 1.40 | 2.91 ± 1.51 | <0.001 | 3.39 ± 1.44 | 3.01 ± 1.46 | 2.84 ± 1.51 | <0.001 | 3.18 ± 1.44 | 3.08 ± 1.49 | 2.92 ± 1.53 | 0.293 |

**Continue.**

| **Factor or Statement** | Residence type | | | Age | | | |
| --- | --- | --- | --- | --- | --- | --- | --- |
|  | Urban areas | Non-urban areas | p | ≤ 30 | 31-50 | ≥ 51 | p |
| **Knowledge** |  |  |  |  |  |  |  |
| The basics of photoaging (K1-K3) | 2.35 ± 0.96 | 2.19 ± 1.04 | 0.095 | 2.15 ± 1.14 | 2.39 ± 0.91 | 2.39 ± 0.82 | 0.007 |
| UV and Sun Protection (K4-K10) | 3.19 ± 1.70 | 3.06 ± 1.79 | 0.428 | 3.06 ± 1.83 | 3.25 ± 1.66 | 2.91 ± 1.69 | 0.160 |
| Methods to improve photoaging (K11-K12) | 0.81 ± 0.68 | 0.79 ± 0.62 | 0.725 | 0.80 ± 0.65 | 0.84 ± 0.68 | 0.54 ± 0.61 | 0.002 |
| **Attitude** |  |  |  |  |  |  |  |
| Anxiety due to skin aging caused by photoaging (A2) | 2.20 ± 1.16 | 2.26 ± 1.19 | 0.623 | 2.39 ± 1.21 | 2.17 ± 1.13 | 1.88 ± 1.11 | 0.004 |
| Sun protection is not only to be anti-aging but also to fight cancer (A4) | 4.46 ± 0.94 | 4.64 ± 0.77 | 0.053 | 4.60 ± 0.84 | 4.40 ± 0.98 | 4.76 ± 0.58 | <0.001 |
| Sun protection is needed not only in summer but all year round (A5) | 4.60 ± 0.80 | 4.71 ± 0.68 | 0.151 | 4.71 ± 0.65 | 4.56 ± 0.84 | 4.81 ± 0.68 | 0.005 |
| Although I can accept natural aging, I still think it is important to prevent photoaging (A6) | 4.61 ± 0.73 | 4.70 ± 0.70 | 0.210 | 4.67 ± 0.64 | 4.57 ± 0.79 | 4.91 ± 0.29 | <0.001 |
| Sunlight can replenish vitamin D. It can also replenish calcium and prevent and improve osteoporosis, so sun protection is not necessary (A8) | 3.17 ± 1.57 | 3.64 ± 1.46 | 0.002 | 3.49 ± 1.58 | 3.17 ± 1.53 | 2.94 ± 1.71 | 0.009 |
| **Practice** |  |  |  |  |  |  |  |
| Reduce skin exposure and use sunscreen products (P1.1-P1.5) | 16.44 ± 5.75 | 18.06 ± 5.82 | 0.004 | 17.93 ± 5.85 | 16.06 ± 5.82 | 17.30 ± 4.43 | <0.001 |
| Routine use of antioxidants (P2) | 2.62 ± 1.55 | 3.02 ± 1.58 | 0.008 | 3.04 ± 1.62 | 2.52 ± 1.53 | 2.72 ± 1.45 | <0.001 |
| Topical application of appropriate concentrations of retinoic acid (P3) | 2.27 ± 1.51 | 2.53 ± 1.57 | 0.085 | 2.73 ± 1.65 | 2.14 ± 1.44 | 2.21 ± 1.45 | <0.001 |
| Chemical peel agent treatment (P4) | 2.85 ± 1.47 | 3.22 ± 1.47 | 0.010 | 3.30 ± 1.47 | 2..75 ± 1.46 | 2.81 ± 1.41 | <0.001 |
| Laser and intense pulsed light (P5) | 2.99 ± 1.50 | 3.48 ± 1.46 | <0.001 | 3.41 ± 1.53 | 2.92 ± 1.47 | 2.94 ± 1.43 | <0.001 |
| Radiofrequency microneedling (P6) | 2.94 ± 1.50 | 3.40 ± 1.43 | 0.002 | 3.34 ± 1.54 | 2.87 ± 1.46 | 2.99 ± 1.42 | <0.001 |
| Mesotherapy (P7) | 3.01 ± 1.49 | 3.35 ± 1.45 | 0.022 | 3.37 ± 1.53 | 2.91 ± 1.46 | 3.22 ± 1.42 | <0.001 |
